# Neuroprotective effect of normal and modified mesenchymal stem cell‐derived exosomes by mitigating Alzheimer's‐related oxidative and inflammatory damage via Nrf2/HO‐1 in SH‐SY5Y cells

**DOI:** 10.1002/ibra.70027

**Published:** 2026-08-09

**Authors:** Rishika Dhapola, Prajjwal Sharma, Sneha Kumari, Balachandar Vellingiri, Dibbanti HariKrishnaReddy

**Affiliations:** ^1^ Advanced Pharmacology and Neuroscience Laboratory, Department of Pharmacology, School of Health Sciences Central University of Punjab, Ghudda Bathinda Punjab India; ^2^ Neurobiology (Ageing and Pediatric) Laboratory, Department of Zoology, School of Basic Sciences Central University of Punjab Bathinda Punjab India

**Keywords:** Alzheimer's disease, exosomes, inflammation, oxidative stress, SH‐SY5Y cells

## Abstract

Exosome therapy is emerging as a promising neuroprotective strategy for Alzheimer's disease (AD). We evaluated and compared whether normal exosomes (NE) and modified exosomes (ME) derived from AD rat brain extract‐treated rat bone marrow mesenchymal stem cells possess the potential to protect SH‐SY5Y cells against streptozotocin (STZ) induced toxicity. The effect of exosomes on oxidative stress, inflammation and neuronal survival was evaluated. Further, antioxidant mechanism of exosomes by nuclear factor erythroid 2‐related factor 2/heme oxygenase‐1 (Nrf2/HO‐1) signaling was explored. Cells were exposed to 5 mM STZ and treated with NE and ME at an equivalent concentration of 50 µg/mL. Exosomes were characterized by specific exosomal markers, CD63 and CD9. Cell viability was assessed using the MTT assay. Neuronal growth and survival were evaluated by measuring brain‐derived neurotrophic factor (BDNF) using ELISA and neuronal nuclei (NeuN) expression using immunocytochemistry. Intracellular reactive oxygen species (ROS) levels were determined using H_2_DCFDA, while inflammatory mediators, including interleukin‐6 (IL‐6) and tumor necrosis factor‐α (TNF‐α) were quantified by ELISA. Expression levels of Nrf2 and HO‐1 were also determined. Exosome treatment improved cell viability, reduced ROS, and lowered IL‐6 and TNF‐α levels. Immunocytochemistry quantification showed increased nuclear Nrf2 and HO‐1 expression in exosome‐treated cells. Moreover, our data indicate that ME derived from AD rat brain extract‐treated rat bone marrow mesenchymal stem cells are more potent in protecting SH‐SY5Y cells from STZ‐induced oxidative stress and inflammation, possibly via Nrf2/HO‐1 signaling, as compared to NE. These in vitro results support further preclinical evaluation of exosome‐based strategies for AD.

## Graphical abstract

Exosome therapy shows promise in Alzheimer's disease. This study compared normal and modified exosomes from AD brain extract‐treated MSCs in STZ‐induced SH‐SY5Y cells. Exosomes improved neuronal viability, reduced oxidative stress and inflammation, and activated Nrf2/HO‐1 signaling. Modified exosomes showed superior neuroprotection, supporting further preclinical investigation.

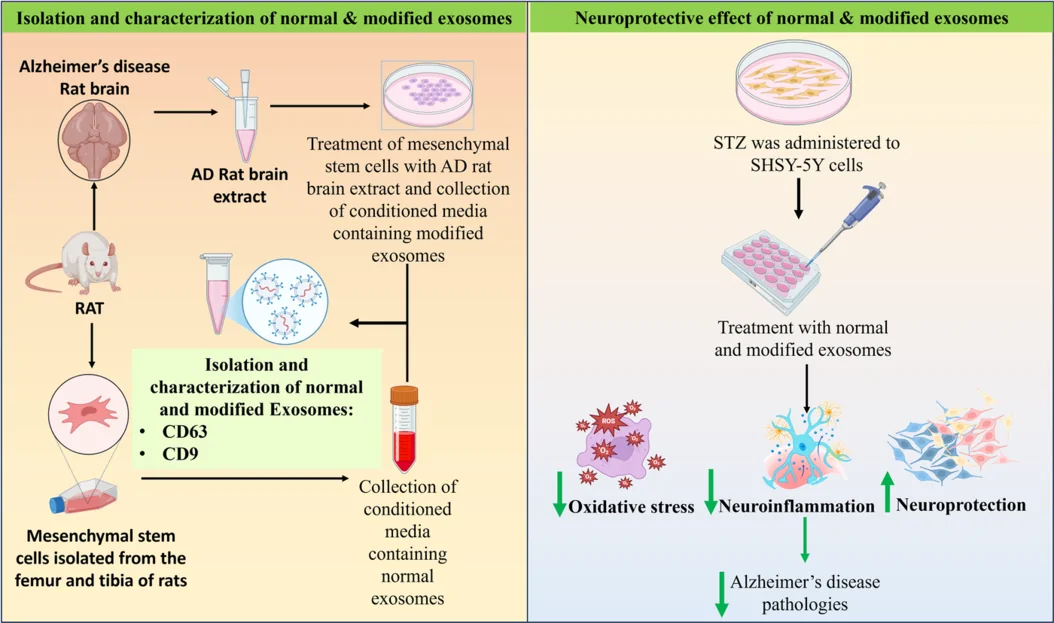

## 1 INTRODUCTION

Alzheimer's disease (AD) is a multifactorial neurodegenerative disorder and the leading cause of dementia worldwide, characterized by progressive cognitive decline, synaptic dysfunction, and neuronal loss.^1^, ^2^ While amyloid‐β (Aβ) aggregation and tau hyperphosphorylation remain defining pathological hallmarks, growing evidence identifies oxidative stress and sustained neuroinflammation as critical upstream drivers of neuronal damage and disease progression. These processes precede overt plaque and tangle formation and actively exacerbate Aβ toxicity, tau pathology, and mitochondrial dysfunction, positioning them as strategic therapeutic targets in AD.^3^, ^4^

Neurons are particularly vulnerable to oxidative stress due to their high metabolic demand and limited regenerative capacity. In AD, excessive ROS generation, impaired antioxidant defense systems, and mitochondrial dysfunction result in apoptotic cell death. Parallel to this, chronic neuroinflammation mediated by glial activation and increased production of pro‐inflammatory cytokines amplifies oxidative injury and disrupts neuronal homeostasis.^5^ The nuclear factor erythroid 2‐related factor (Nrf2) signaling pathway is a major regulator of cellular redox balance by inducing heme oxygenase‐1 (HO‐1), an antioxidant and cytoprotective gene. Notably, impaired Nrf2 activity has been reported in AD, contributing to heightened oxidative burden and inflammatory signaling.^6^, ^7^

Exosomes, nanosized extracellular vesicles (EVs) (30–150 nm), have emerged as potent modulators of neurobiological processes through their ability to transfer functional biomolecules such as proteins, lipids, and regulatory RNAs between cells.^8^, ^9^ Owing to their intrinsic stability, lesser immunogenicity, and capacity to penetrate the blood–brain barrier, exosomes offer distinct advantages over conventional pharmacological and cell‐based therapies. Recent evidence suggests that exosomes can attenuate oxidative stress, suppress inflammatory cascades, and enhance neuronal survival; however, their mechanistic role in regulating oxidative stress and inflammation interplay in neuronal models of AD remains insufficiently explored.^10^

Studies suggest that if the protein content of these exosomes is enhanced, they can exhibit more potent therapeutic activity against AD. In accordance with a study involving rat brain extract, it was observed that EVs derived from mesenchymal stem cells (MSCs), which were treated with brain‐extract, possessed high levels of the growth factors, including brain‐derived neurotrophic factor (BDNF), nerve growth factor, vascular endothelial growth factor, and hepatocyte growth factor, as compared to normal EV.^11^ Further, brain extract‐treated MSC‐EVs were found to reduce neuroinflammation and enhance neurogenesis and angiogenesis.^12^ In our study, we utilized the SH‐SY5Y cell line to mimic AD pathologies. We investigated the therapeutic efficacy of normal exosomes (NE) and modified exosomes (ME) and compared their potential in counteracting oxidative stress and neuroinflammation. We evaluated the effects of NE and ME on ROS production, inflammatory mediators, and neuronal integrity, with particular emphasis on modulation of the Nrf2/HO‐1 antioxidant pathway. By delineating the molecular mechanisms underlying exosome‐mediated neuroprotection, this work aims to strengthen the translational relevance of exosome‐based interventions targeting oxidative stress and inflammation in AD.

## 2 MATERIALS AND METHODS

### 2.1 Reagents

Total exosome isolation reagent (from cell culture media) (Himedia, Cat. No. 4478359), Dulbecco's modified Eagle medium (DMEM) (Himedia, Cat. No. AL007A) high glucose, fetal bovine serum (FBS) (Himedia, Cat. No. RM9955), antibiotic solution (Himedia, Cat. No. A001A), phosphate buffer saline (PBS) (Himedia, Cat. No. TL1006), trypsin‐EDTA 0.25% (Himedia, Cat. No. TCL007), CD63 (Affinity Biosciences, Cat. No. AF5117; AB_2837603; 1:200) and CD9 (Affinity Biosciences, Cat. No. AF5139; AB_2837625; 1:200) antibodies, Alexa Fluor‐647 (G‐Biosciences, Cat. No. ITIF64711; 1:500), streptozotocin (Himedia, Cat. No. MB284), 3‐(4,5‐dimethylthiazol‐2‐yl)−2,5‐diphenyltetrazolium bromide (MTT), and dimethyl sulfoxide (DMSO) anhydrous, were obtained from Invitrogen Pvt. Ltd. Other reagents include BDNF (Elabsciences, Cat. No. E‐EL‐H0010), tumor necrosis factor‐α (TNF‐α) (G‐Biosciences, Cat. No. ITLK01164) and interleukin‐6 (IL‐6) (G‐Biosciences, Cat. No. ITLK02419) ELISA kits, NeuN antibody (G‐Biosciences, Cat. No. ITT06145; 1:200), Nrf2 antibody (Affinity Biosciences, Cat. No. AF0639; AB_2833793; 1:200), polyclonal antibody to HO‐1 (Cloud clone corp, Cat. No. PAA584Hu02; 1:200), 2,7' Dichlorofuoroescin diacetate (H_2_DCFDA), and DAPI (Himedia, Cat. No. D9542).

### 2.2 Animals

10 Wistar rats (M/F) around 180–270 g were obtained from Lala Lajpat Rai University of Veterinary and Animal Sciences, Hisar. Five rats were used for MSCs isolation and five were used to collect brain extract. All the rats were maintained in the animal house facility of Central University of Punjab, Bathinda, India. The animals were housed in a room with a 12‐h light/dark cycle and had unrestricted access to food and water. Every experiment was carried out in compliance with institutional policies for the care and management of animals, and the institutional animal ethics committee (IAEC) of Central University of Punjab authorized the study protocol (IAEC approval no. CUPB/IAEC/2022/53).

### 2.3 Collection of AD rat brain extract

Rats were injected with ketamine and xylazine intraperitoneally to anesthetize them in 80 mg/kg and 10 mg/kg doses, respectively. Midline sagittal incision was made on the scalp of the rats using the stereotaxic apparatus. Streptozotocin (STZ) was administered intracerebroventricularly (ICV) to induce AD. The stereotaxic co‐ordinates employed were 3.6 mm dorsoventral, 0.8 mm anteroposterior, and 1.5 mm mediolateral according to the bregma. 3 mg/kg freshly prepared STZ was administered bilaterally.^13^, ^14^ Seven days after receiving STZ, rats with compromised cognitive abilities were sacrificed. The evaluation of impaired cognitive function in the rats was done using the neurobehavioral tests. Later, the rats were sacrificed using proper anesthesia followed by cervical dislocation, and brain was isolated. Homogenate was prepared by using rat brain tissue slices and DMEM (150 mg/mL). Homogenates of the tissue samples were subjected to two rounds of centrifugation for 60 min at 10,000 rpm at 4°C and subsequently passed through a 0.2 μm filter to minimize residual contaminants. Total protein level was quantified by Lowry's method. The resultant brain extract supernatant was kept at −80°C until further use.^12^

### 2.4 Isolation of MSCs from rat bone marrow

Wistar rats (2 months old) (*n* = 5) were sedated using ketamine and xylazine, 80 mg/kg and 10 mg/kg, respectively, and sacrificed by cervical dislocation. The tibias were dissected to obtain bone marrow MSCs (BMSCs). The suspension of the cells was subjected to centrifugation at 1000 × *g* for 5 min, and cell pellets were subsequently harvested and dispersed in complete medium, consisting of DMEM, 10% FBS, and 1% antibiotic solution. The cells adhering to the culture flask were considered as P0 (Passage 0) BMSCs; 2 days later, complete media was changed. Trypsin containing ethylenediaminetetraacetic acid was used to digest the cells at 80–90% confluency for 1 to 2 min at 37°C. The cell suspension was reseeded into another flask, once the digestion was stopped with the complete medium. Exosomes were isolated from the conditioned media of BMSCs at passages 3 to 6.

### 2.5 Isolation of rat BMSC‐derived normal and modified exosomes

#### 2.5.1 Normal exosomes

The conditioned media from BMSCs of 3rd−6th passage was collected and subjected to centrifugation at 10,000 × *g* for 30 min to exclude cells and debris. Exosomes were then derived by precipitation method using the total exosome isolation reagent (from cell culture media) in accordance with the protocol provided by the manufacturer. Supernatant was removed and the pellet was resuspended in PBS and kept at −80°C until further analysis. Each of these processes was carried out at 4°C.

#### 2.5.2 Modified exosomes

The BMSCs from 3rd−6th passage were cultured with medium containing 10% of AD rat brain extract to stimulate the cells to produce modified exosomes. Conditioned media containing exosomes was collected from BMSCs at 80–90% confluency after 24 h of brain extract treatment. Modified exosomes were isolated using total exosome isolation reagent and stored in aliquots at −80°C until further use.^15^ Before use, the exosomes were first transferred to −20°C, followed by 4°C to reduce stress due to sudden changes in temperature.

### 2.6 Characterization and cargo assessment of rat BMSCs‐derived normal and modified exosomes

Flow cytometry was used to confirm the exosomal origin of vesicles obtained from rat BMSCs. Exosomes were incubated with primary antibodies to tetraspanins CD63 and CD9, which are standard exosomal markers. After primary antibody incubation, samples underwent treatment with goat anti‐rat IgG conjugated to secondary antibody Alexa Fluor‐647, which allowed fluorescence‐based detection. The fluorescent signal corresponding to Alexa Fluor‐647 was recorded using a flow cytometer (Attune cytometric software v5.1.1), and data were analyzed to determine the expression of CD63 and CD9 on the exosome surface, thereby verifying the identity of rat BMSCs‐derived exosomes.^16^ To quantify the total protein content, Lowry's protein estimation method was used. Additionally, BDNF levels were evaluated in the exosome preparations using a commercial ELISA kit. This method allowed for the sensitive and quantitative detection of the level of neurotrophic proteins in the ME as compared to the NE. When taken as a whole, these analyses verified the existence of important bioactive components in the ME as well as the enhanced total protein load in comparison to the NE.

### 2.7 Cell culture

SH‐SY5Y cell line was purchased from the National Center for Cell Science (NCCS), Pune, India, in March 2023, with a complete cell line authentication certificate provided by the Cell Bank at the time of procurement. Furthermore, the quality of the cell lines was authenticated via short tandem repeat (STR) matching analysis by NCCS Pune in March, 2023. All cells utilized in this study were confirmed to be free of mycoplasma contamination. The SH‐SY5Y cells were cultured in DMEM supplemented with high glucose, 10% FBS, and 1% antibiotic solution. The cells were kept at 37°C in a 5% CO_2_ atmosphere with saturated humidity in a CO_2_ incubator. When the cells reached around 80% confluence, they were subcultured using trypsin to separate them from the culture plate surface. Then, at a density of 1 × 10^5^ cells/mL in 24‐well plates or 1 × 10^4^ cells/mL in 96‐well plates, cells were seeded for various experiments. Additionally, the remaining cells were maintained in T‐25 (25 cm^2^) and T‐75 (75 cm^2^) culture flasks to support continued growth and expansion.^17^

### 2.8 Dose selection

The dose of STZ was chosen based on previous literature reports. Prior studies have indicated that treatment with STZ at a dose of 5 mM for 24 h significantly induces inflammation, oxidative stress and cellular damage.^18^, ^19^ Similarly, the exosome dose of 50 µg/mL was chosen based on published research showing that this dosage had notable therapeutic effects. It has been demonstrated that this dosage improves pathogenesis while preserving an adequate safety profile.^20^ Also, MTT was performed in our study to support the therapeutic potential of exosomes at 50 µg/mL dose. Based on previous literature and MTT assay, we selected this dose for further studies.

### 2.9 Measuring cell viability

The neuroprotective activity of NE and ME was assessed in terms of formazan generated using MTT in STZ‐treated SH‐SY5Y cells. Briefly, the cells (1 × 10^4^ cells/well) in 96‐well plates were subjected to 10, 20, and 50 µg/mL of NE and 10, 20, and 50 µg/mL of ME for the next 24 h, and the absorbance of each sample was measured at 570 nm using the Spark® Multimodal Microplate reader.^21^

### 2.10 ELISA

In a six‐well plate, SH‐SY5Y cells were seeded at a density of 3 × 10^5^ cells/well. Then the cells were treated with 5 mM STZ for 24 h, followed by 50 µg/mL NE, or 50 µg/mL ME for the next 24 h. Then, the cells were rinsed with PBS and undergone centrifugation at 14,000 *g* for 30 min. After centrifugation, the supernatants were transferred to newly labeled tubes. BDNF, TNF‐α, and IL‐6 levels were quantified using the corresponding ELISA kit following the manufacturer's instructions, with absorbance measured at 450 nm.^22^

### 2.11 Measuring intracellular ROS level

The SH‐SY5Y cells (1 × 10^4^ cells/well) were seeded in a 96‐well plate and treated with STZ 5 mM at 37°C, 5% CO_2_ for 24 h. The cells were then treated with 50 µg/mL NE or 50 µg/mL ME for another 24 h. The fluorescence intensity was measured at 485 nm for absorbance and 535 nm for emission after incubating the cells with H_2_DCFDA (10 μM) for 30 min using a Spark® Multimodal Microplate Reader.^23^, ^24^

### 2.12 Immunocytochemistry

The monolayer of the SH‐SY5Y cells (1 × 10^5^ cells/well) was seeded on the coverslip^25^ and treated with STZ 5 mM and thereafter treated with 50 µg/mL NE or 50 µg/mL ME at 37°C, 5% CO_2_ as per standard protocol. Subsequently, immunostaining was executed by incubating with anti‐neuN (1:200), anti‐Nrf2 (1:200), and anti‐HO‐1 (1:200) overnight at 4°C, followed by Alexa Fluor 647, at a dilution of 1:500 for 1 h at room temperature. The cells were rinsed twice with PBS. DAPI (1 µg/mL) was used for counterstaining for 5 min, and observed using a confocal laser scanning microscope (Olympus Fluoview Ver.4.2a). All images were analyzed in ImageJ 1.53e software using a fixed threshold applied uniformly to all groups to identify positive staining. Three randomly selected fields were analyzed per well, with three wells evaluated per experimental group.

### 2.13 Statistical analysis

All the experimental data were assessed using GraphPad Prism 8.0.1. Data were represented as mean ± SEM. Significance differences among groups were determined by unpaired two‐tailed Student's *t*‐test and one‐way ANOVA followed by Tukey post hoc test accordingly. Differences were interpreted statistically significant at *p* < 0.05.

## 3 RESULTS

### 3.1 Confirmation of exosomal markers and cargo assessment in normal and modified exosomes

Flow cytometric analysis demonstrated that approximately 80% of the total population in each sample was positive for the exosomal surface markers CD63 and CD9, indicating that the isolated vesicles were predominantly exosomes (Figure 1A). However, due to the inherent limitations of precipitation‐based isolation techniques, co‐isolation of other EVs and protein contaminants cannot be ruled out, despite the enrichment of CD63‐ and CD9‐positive exosomes. Protein enrichment during the isolation process was confirmed by Lowry's assay, which revealed that the ME had a substantially greater total protein concentration than the NE (Figure 1B). Further, BDNF levels were significantly higher in the ME as compared with NE, suggesting effective loading or selective preservation of this neurotrophic factor. When taken as a whole, our results confirm the enrichment of functionally important molecules like BDNF in the isolated ME as well as the total protein enhancement in comparison with the NE (Figure 1C).

**Figure 1:**
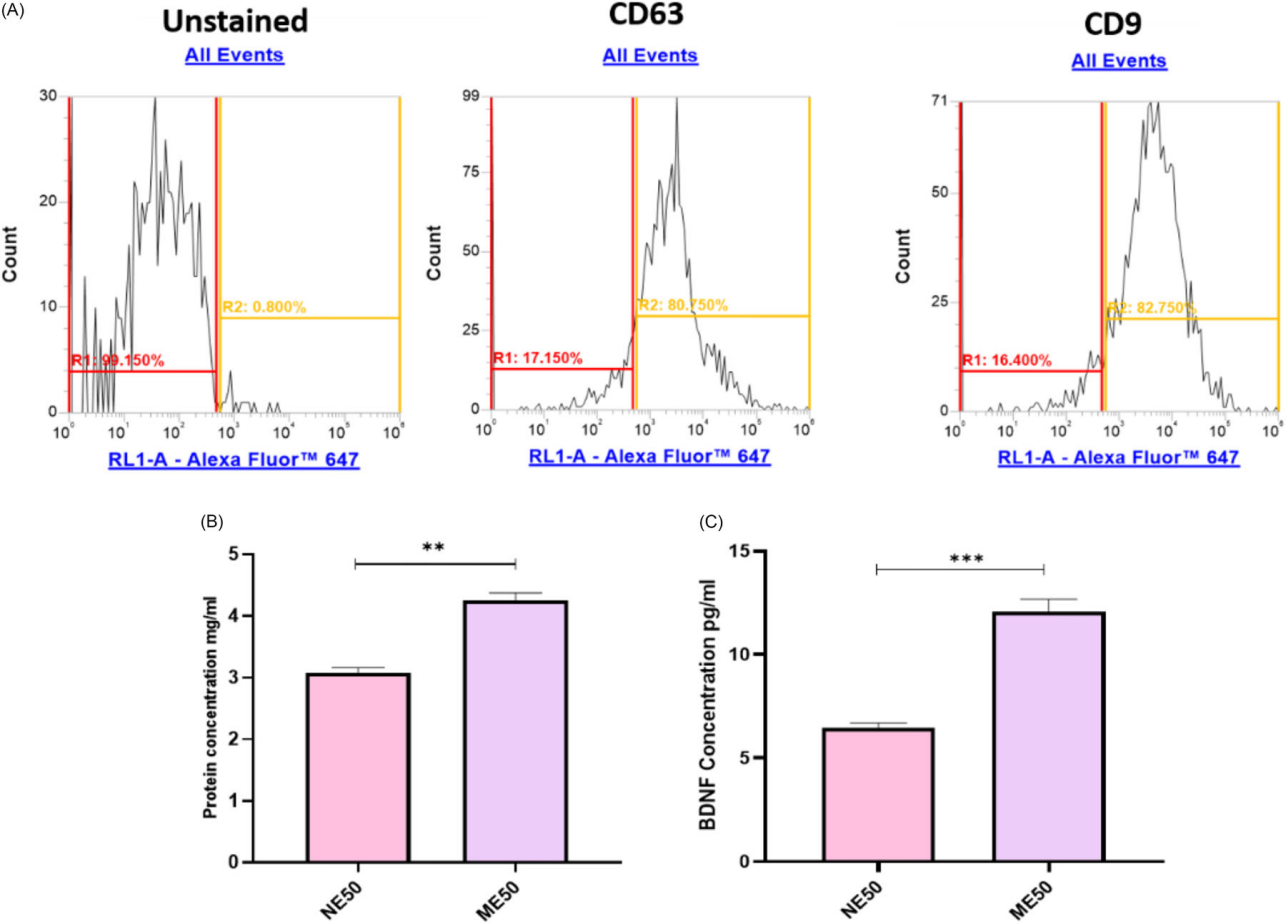
Characterization of rat BMSC‐exosomes. (A) Flow cytometry examination of rat BMSC‐exosomes using exosome markers CD63 and CD9 antibodies. Histograms demonstrate the number of counts incubated with the specific antibody. The logarithm on the *x*‐axis indicates the strength of the fluorescent signal, while the *y*‐axis shows the number of counts. Exosomes isolated from rat BMSCs were positive for the markers CD63 and CD9, and approximately 80% of the total counts in each sample expressed exosomal markers, suggesting the presence of desired exosomal population. (B) Lowry's assay revealed a significant increase in total protein concentration in ME samples compared with NE (**p = 0.0017). (C) ELISA quantification showed elevated BDNF levels in ME than in NE (***p = 0.0009), confirming the presence of key neurotrophic proteins within the modified exosomal preparations. Data are presented as mean ± SEM (*n* = 3). Statistical analysis was performed using an unpaired two‐tailed Student's *t*‐test; *p* < 0.05 was considered statistically significant. ME, modified exosomes; NE, normal exosomes.

### 3.2 Effect of exosomes on SH‐SY5Y cells

STZ treatment significantly reduced the viability of SH‐SY5Y cells. Treatment with lower concentrations of exosomes (STZ + NE10, STZ + NE20, STZ + ME10, and STZ + ME20) did not produce any significant improvement in cell viability, indicating limited neuroprotective effects at these doses. In contrast, higher concentrations of exosomes (STZ + NE50 and STZ + ME50) resulted in enhanced cell viability, with the STZ + ME50 group demonstrating a significantly greater protective effect (Figure 2).

**Figure 2:**
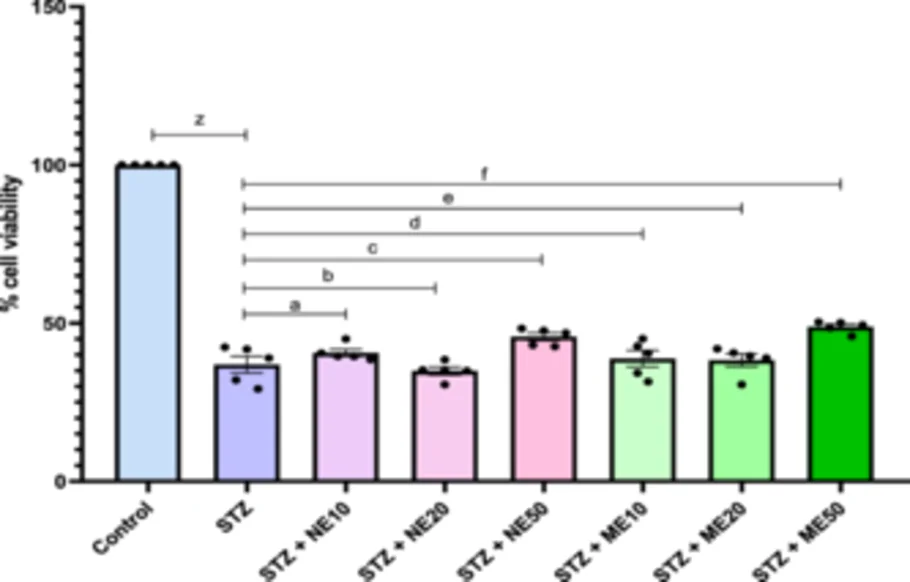
NE and ME at 50 µg/mL increased cell viability. Determination of cell viability of NE and ME at different concentrations (10, 20, and 50 µg/mL) in SH‐SY5Y cells: the viability of the cells in the ME50 group was significantly higher compared to the STZ‐treated cells. All values are represented as mean ± SEM (*n* = 5). Statistical analysis was carried out using one‐way ANOVA followed by Tukey's post hoc test. ^*z*^ *p* < 0.0001 (Control vs. STZ); ^*a*^ *p *= non‐significant (0.7615) (STZ vs. STZ + NE10); ^*b*^ *p *= non‐significant (0.9890) (STZ vs. STZ + NE20); ^*c*^ *p* = 0.061 (STZ vs. STZ + NE50); ^*d*^ *p *= non‐significant (0.9915) (STZ vs. STZ + ME10); ^*e*^ *p *= non‐significant (0.9985) (STZ vs. STZ + ME20); ^*f*^ *p* = 0.0004 (STZ vs. STZ + ME50). ME, modified exosomes; NE, normal exosomes; STZ, streptozotocin.

### 3.3 Effect of exosomes on the level of neurotrophic factors

We investigated whether exosome treatment elevated the level of neurotrophic factor, BDNF, in STZ‐treated SH‐SY5Y cells. As shown in Figure 3, BDNF levels were suppressed in the STZ‐treated group in comparison with the control group (^*a*^ *p* < 0.0001). Conversely, this reduction in BDNF was ameliorated upon exosome treatment that significantly preserved BDNF levels. Further, ME showed increased potential in restoring BDNF levels as compared to NE (^*c*^ *p* < 0.0132). Therefore, our results substantiate that exosome treatment at a dose of 50 µg/mL significantly increases the levels of BDNF.

**Figure 3:**
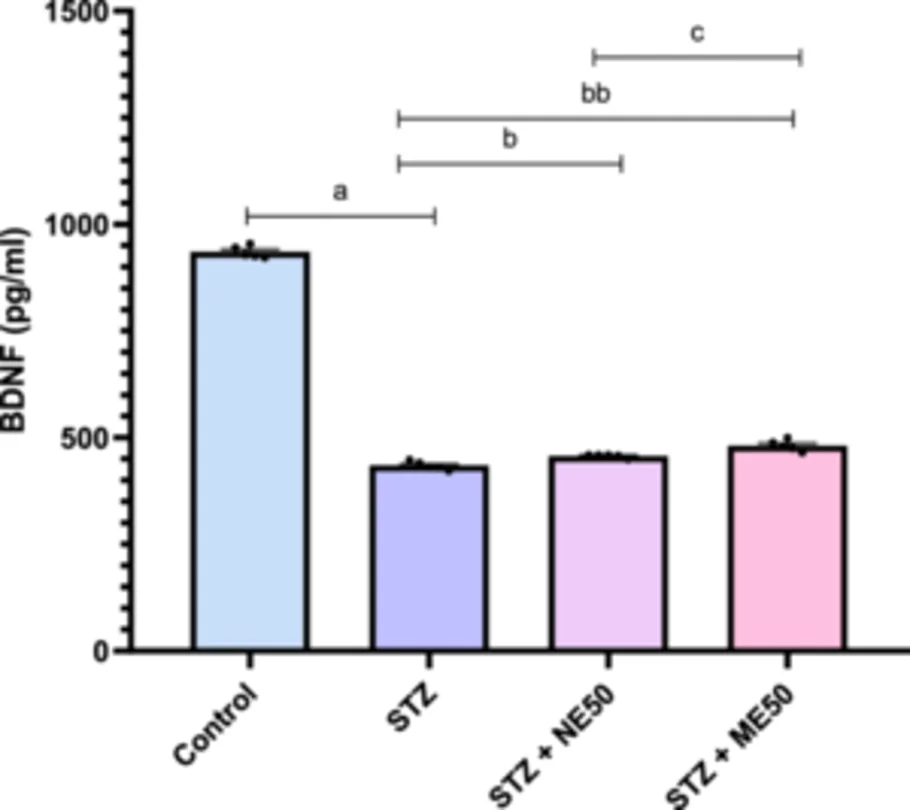
BDNF levels were increased upon exosome treatment. Cells were exposed to 5 mM STZ, and the treatment groups STZ + NE50 and STZ + ME50 received 50 µg/mL of NE and ME, respectively. All values are presented as mean ± SEM (*n* = 5). Statistical analysis was carried out using one‐way ANOVA followed by Tukey's post hoc test. Different letters indicate significant differences: ^*a*^ *p* < 0.0001 (control vs. STZ), ^*b*^ *p* < 0.0226 (STZ vs. STZ + NE50); ^*bb*^ *p* < 0.0001 (STZ vs. STZ + ME50), and ^*c*^ *p* < 0.0132 (STZ + NE50 vs. STZ + ME50). BDNF, brain‐derived neurotrophic factor; ME, modified exosomes; NE, normal exosomes; STZ, streptozotocin.

### 3.4 Effect of exosomes on neuronal survival

Immunocytochemical analysis was performed to examine the expression of NeuN, a neuronal nuclear protein, in SH‐SY5Y cells across control, STZ, NE, and ME‐treated groups. In the control group, strong NeuN immunoreactivity was observed, indicating a healthy neuronal status. In contrast, the STZ‐treated group showed a significant reduction in NeuN fluorescence intensity (^*a*^ *p* < 0.0001), suggesting neuronal damage or loss induced by neurotoxic stress. Treatment with NE 50 µg/mL (^*b*^ *p* = 0.0101), and ME 50 µg/mL (^*bb*^ *p* < 0.0001) markedly restored NeuN expression, as demonstrated by increased fluorescence intensity compared to the STZ group (Figure 4). Further, it was observed that ME significantly increased the expression of NeuN as compared to NE (^*c*^ *p* = 0.0306). These results indicate that exosomes potentially protect the cells against STZ‐induced toxicity.

**Figure 4:**
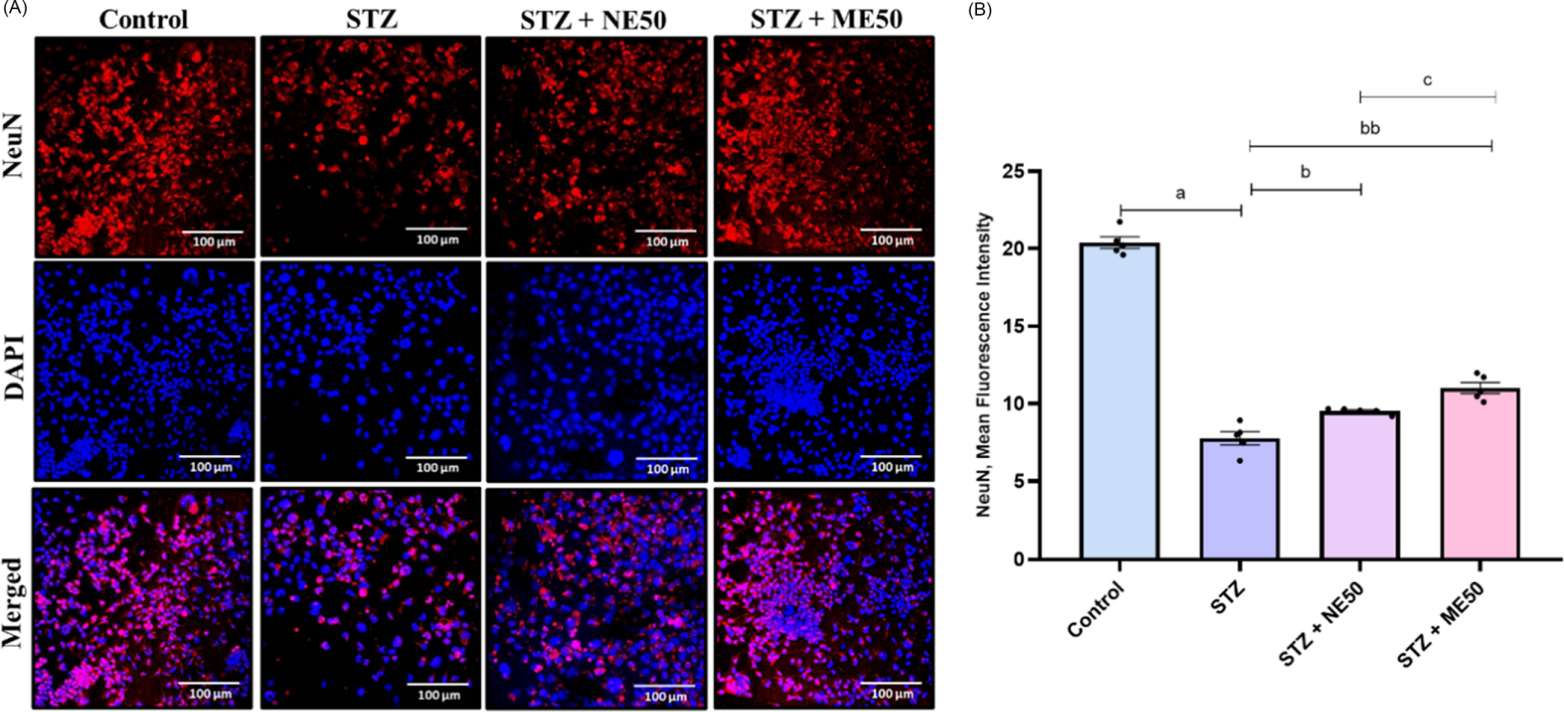
Effects of exosomes on neuronal survival. (A) Exosomes increased neuronal cell survival as evidenced by increased NeuN expression. STZ exposure reduced NeuN immunoreactivity, confirming neuronal damage. Treatment groups showed restored NeuN expression, with ME 50 µg/mL (STZ + ME50) exhibiting the highest expression (20× magnification). (B) NeuN mean fluorescence intensity (MFI) was quantified using ImageJ. STZ significantly reduced MFI, while exosomes increased NeuN expression relative to STZ. Further, the expression of NeuN is higher in the ME‐treated group in comparison to the NE‐treated group. Data are presented as mean ± SEM. One‐way ANOVA followed by Tukey's post hoc test was used for all analyses. The letters indicate significant differences: ^*a*^ *p* < 0.0001 (control vs. STZ), ^*b*^ *p* = 0.0101 (STZ vs. STZ + NE50); ^*bb*^ *p* < 0.0001 (STZ vs. STZ + ME50) and ^*c*^ *p* = 0.0306 (STZ + NE50 vs. STZ + ME50); (*n* = 5). Scale bars: 100 µm. Color codes: DAPI nuclei staining, blue; NeuN staining, red. ME, modified exosomes; NE, normal exosomes; NeuN, neuronal nuclei; STZ, streptozotocin.

### 3.5 Effect of exosomes on intracellular ROS production

We assessed whether exosomes suppressed intracellular ROS generation in STZ‐treated SH‐SY5Y cells using H_2_DCFDA assay. As represented in Figure 5, intracellular ROS was elevated in STZ‐treated cells compared to the control cells (^*a*^ *p* < 0.0001). Inversely, this increased ROS declined upon exosome treatment. Further, ROS levels were more significantly reduced in ME‐treated cells as compared to the NE‐treated cells (^*c*^ *p* = 0.0038). Therefore, our results suggest effective ROS scavenging or antioxidant activity of exosomes.

**Figure 5:**
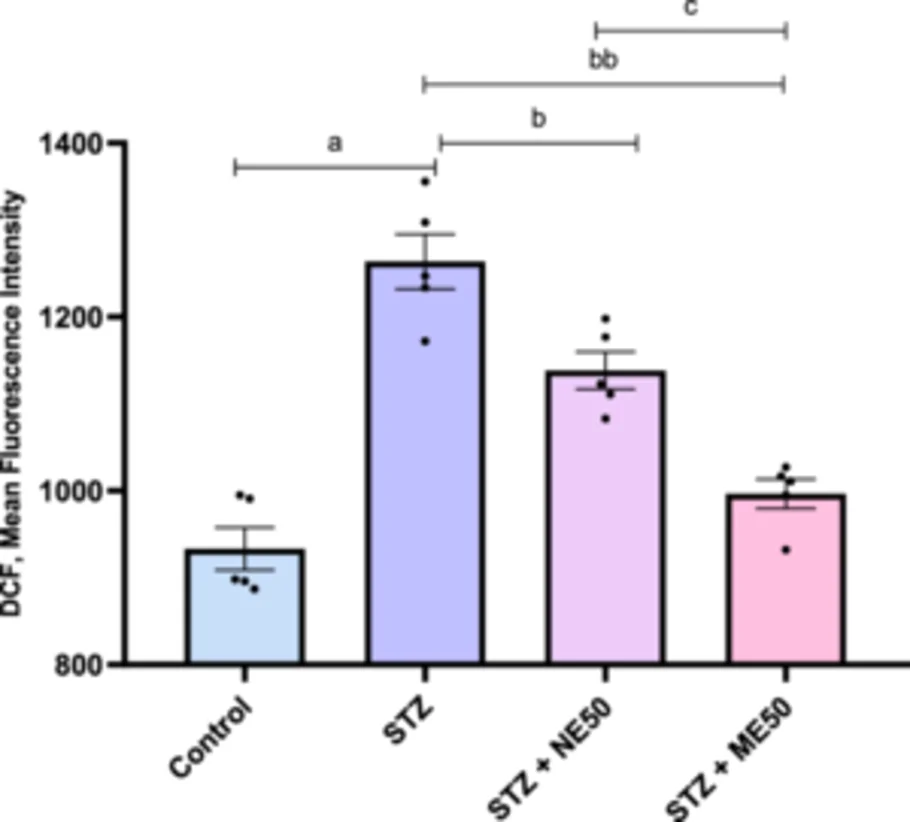
Exosomes suppressed intracellular ROS levels. The H_2_DCFDA assay detected intracellular ROS accumulation in various groups. All values are represented as mean ± SEM (*n* = 5). Statistical analysis was carried out using one‐way ANOVA followed by Tukey's post hoc test. Different letters indicate significant differences: ^*a*^ *p* < 0.0001 (control vs. STZ), ^*b*^ *p* = 0.0103 (STZ vs. STZ + NE50), ^*bb*^ *p* < 0.0001 (STZ vs. STZ + ME50), and ^*c*^ *p* = 0.0038 (STZ + ME50 vs. STZ + NE50). ME, modified exosomes; NE, normal exosomes; STZ, streptozotocin.

### 3.6 Effect of exosomes on Nrf2/HO‐1 expression

We used immunocytochemistry to determine the effect of exosomes on the immunoreactivity of Nrf2/HO‐1. In the control group, moderate nuclear Nrf2 and cytoplasmic HO‐1 immunoreactivity was observed, indicating baseline antioxidant signaling. STZ exposure resulted in a significant reduction in Nrf2 and HO‐1 expression, reflecting oxidative stress and dysregulated antioxidant response. Notably, treatment with NE and ME 50 µg/mL led to a pronounced increase in Nrf2 expression, and a concurrent upregulation of HO‐1 levels, compared to the STZ group (Figure 6A,B). Quantitative fluorescence intensity analysis showed a significant reduction in Nrf2/HO‐1 levels in the STZ group relative to control (*p* < 0.0001). Exosome treatment significantly enhanced both Nrf2 and HO‐1 expression in comparison to the STZ group (*p* < 0.05) (Figure 6C,D). ME more significantly increased Nrf2/HO‐1 expression as compared to NE. Overall, these results indicate the anti‐oxidative effect of exosomes may be via the Nrf2/HO‐1 pathway.

**Figure 6:**
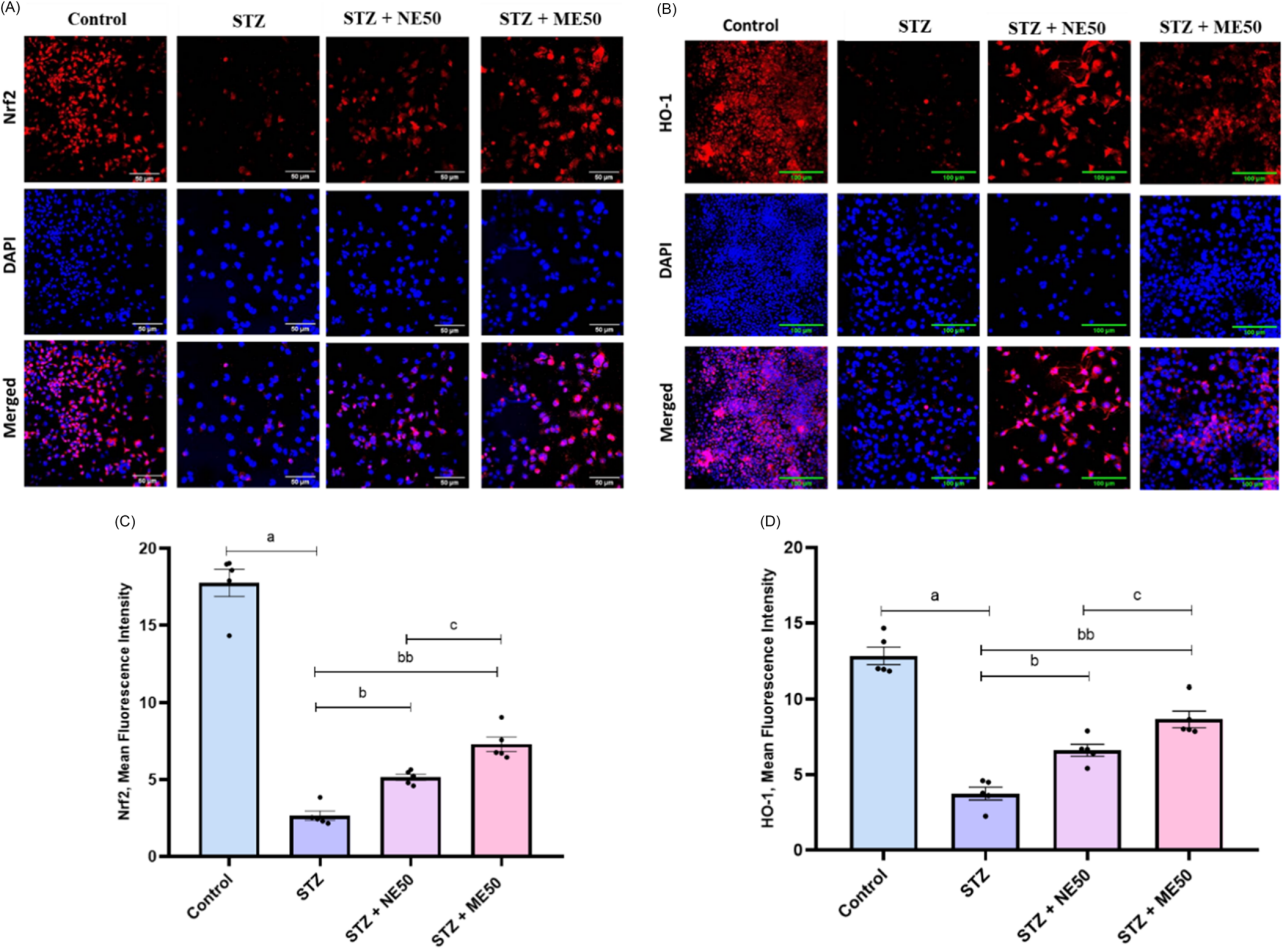
Effect of exosomes on Nrf2/HO‐1 expression. (A, B) Exosomes increased Nrf2 and HO‐1 expression. STZ treatment reduced Nrf2 and HO‐1 immunoreactivity, indicating impaired antioxidant response. Treatment groups restored expression, with ME50 showing the most prominent increase. Images were captured at 20× magnification. (C, D) Quantification of Nrf2 and HO‐1 fluorescence. Mean fluorescence intensities (MFI) were calculated using ImageJ from multiple fields. Data are represented as mean ± SEM. One‐way ANOVA followed by Tukey's post hoc test was used for all analyses. Significance indicators (C): ^*a*^ *p* < 0.0001 (control vs. STZ), ^*b*^ *p* = 0.0208 (STZ vs. STZ + NE50), ^*bb*^ *p* < 0.0001 (STZ vs. STZ + ME50), and ^*c*^ *p* = 0.0493 (STZ + ME50 vs. STZ + NE50); Significance indicators (D): ^*a*^ *p* < 0.0001 (control vs. STZ), ^*b*^ *p* = 0.0040 (STZ vs. STZ + NE50), ^*bb*^ *p* < 0.0001 (STZ vs. STZ + ME50), and ^*c*^ *p* = 0.0436 (STZ + ME50 vs. STZ + NE50). Scale bars: 50 µm (A) and 100 µm (B). Color codes: DAPI nuclei staining, blue; Nrf2 and HO‐1 staining, red. ME, modified exosomes; NE, normal exosomes; STZ, streptozotocin.

### 3.7 Effect of exosomes on pro‐inflammatory cytokine release

We investigated inflammatory cytokine secretion from STZ‐treated SH‐SY5Y cells. ELISA demonstrated that inflammatory cytokines TNF‐α and IL‐6 secretions were significantly elevated in the STZ 5 mM group but suppressed in the exosomes‐treated group (*p* < 0.05), indicating the induction of a strong anti‐inflammatory response upon exosomes treatment. Treatment with NE 50 µg/mL and ME 50 µg/mL significantly attenuated the levels of these pro‐inflammatory cytokines. Among the treated groups, ME at a dose of 50 µg/mL demonstrated the most notable reduction in both IL‐6 and TNF‐α (*p* < 0.0001) (Figure 7).

**Figure 7:**
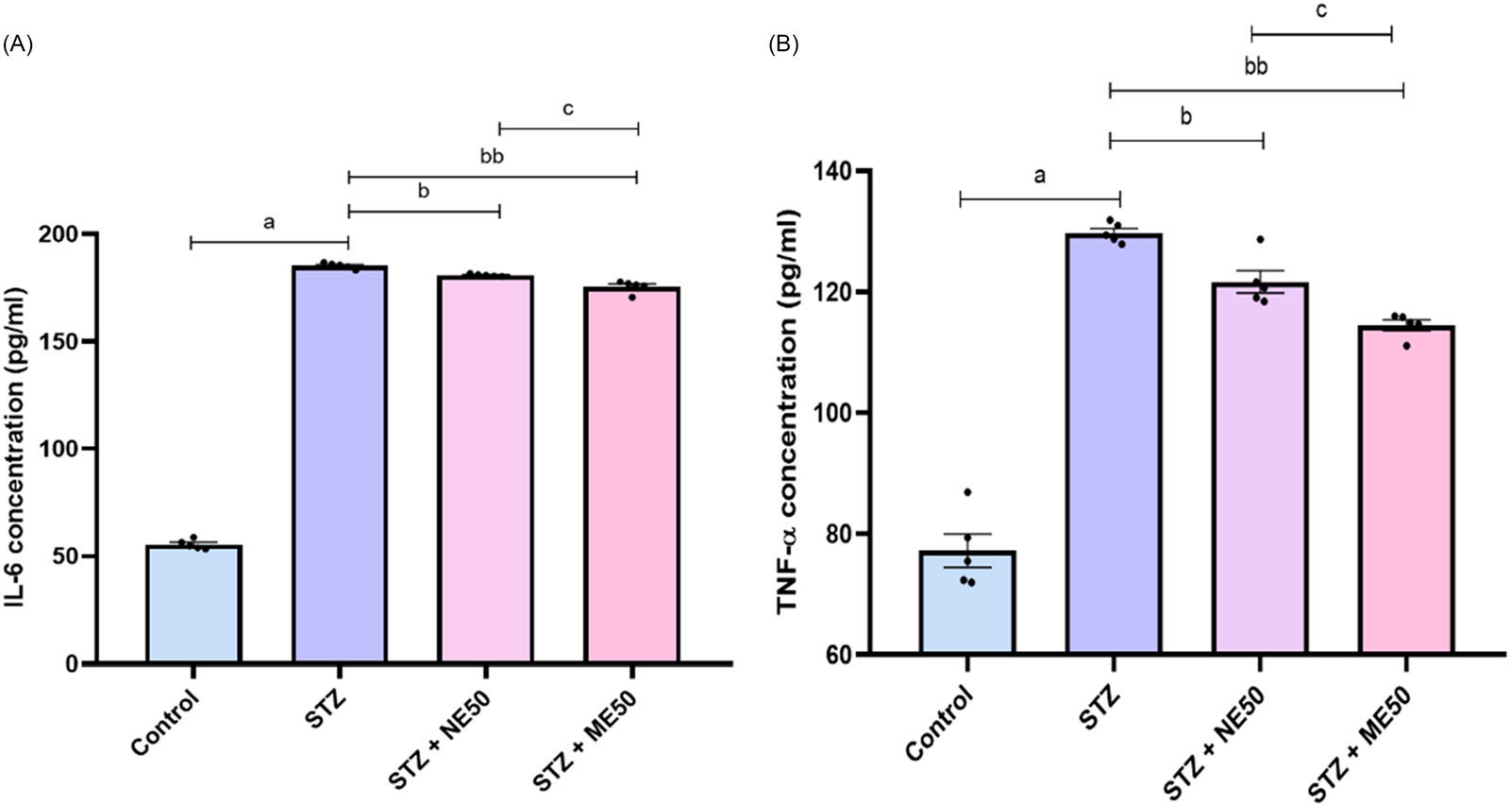
Exosomes mitigated the levels of pro‐inflammatory cytokines. ELISA kits measured the secretion of (A) IL‐6 and (B) TNF‐α. Data are expressed as mean ± SEM (*n* = 5). Different letters indicate significance indicators (A): ^*a*^ *p* < 0.0001 (control vs. STZ), ^*b*^ *p* = 0.0123 (STZ vs. STZ + NE50), ^*bb*^ *p* < 0.0001 (STZ vs. STZ + ME50), and ^*c*^ *p* = 0.0024 (STZ + ME50 vs. STZ + NE50); (B): ^*a*^ *p* < 0.0001 (control vs. STZ), ^*b*^ *p* = 0.0230 (STZ vs. STZ + NE50), ^*bb*^ *p* < 0.0001 (STZ vs. STZ + ME50), and ^*c*^ *p* = 0.0476 (STZ + ME50 vs. STZ + NE50). IL‐6, interleukin‐6; ME, modified exosomes; NE, normal exosomes; STZ, streptozotocin; TNF‐α, tumor necrosis factor‐α.

## 4 DISCUSSION

In this study, we evaluated the neuroprotective activity of rat BMSCs‐derived NE and ME in a STZ‐induced in vitro model of AD using the SH‐SY5Y cell line, with particular emphasis on oxidative stress, neuroinflammation, and neuronal survival. STZ exposure at a concentration of 5 mM induced marked toxicity, as evidenced by reduced cell viability, increased oxidative stress, increased inflammatory responses, and neuronal loss, consistent with earlier reports describing STZ‐mediated mitochondrial dysfunction, ROS overproduction, and neuronal death.^18^ These findings validate the robustness of the STZ‐induced AD model employed in this study.

MSCs are well recognized for their paracrine activity, largely mediated through the secretion of EVs, particularly exosomes. Exosomes are nanosized vesicles (30–150 nm) that regulate intercellular signaling by translocating bioactive cargo, including proteins, lipids, mRNA, and regulatory non‐coding RNAs. Under physiological conditions, they contribute to cellular homeostasis, while under pathological conditions, they can modulate disease‐associated signaling pathways.^26^, ^27^, ^28^ Emerging evidence suggests that disease‐specific cues, including brain‐derived factors present in neurodegenerative conditions, can influence MSCs to release exosomes enriched with neuroprotective components.^12^, ^29^ In line with this concept, our findings support the hypothesis that brain extract‐treated BMSC‐derived ME possess enhanced therapeutic potential against AD‐related neurodegeneration, corroborating previous studies that have highlighted their disease‐modifying capabilities.^12^, ^30^, ^31^, ^32^

MTT assay demonstrated a significant decline in cell viability following STZ treatment, indicative of neuronal injury. Treatment with exosomes significantly restored cell viability, suggesting a robust cytoprotective effect. To further elucidate the effect on neuronal survival, we examined the levels of BDNF, a critical regulator of neuronal survival, synaptic plasticity, and cognitive function.^33^ STZ exposure resulted in a pronounced reduction in BDNF levels, reflecting impaired neuronal maintenance and growth.^34^ Notably, exosome treatment significantly restored BDNF expression, indicating that exosomes may stimulate neuronal repair and survival by preserving neurotrophic cargo. This neuroprotective effect was further supported by the restoration of NeuN expression, a well‐established marker of neuronal integrity^35^ following exosome treatment, which was markedly reduced in STZ‐treated cells. Moreover, the results suggested that treatment with ME resulted in a significant increase in cell viability, BDNF expression, and NeuN levels compared with NE treatment.

In the present study, STZ treatment significantly increased intracellular ROS levels, as assessed by the H_2_DCFDA assay. Exosome treatment markedly attenuated ROS generation, indicating effective mitigation of oxidative damage. Consistent with previous reports demonstrating exosome‐mediated activation of antioxidant pathways.^36^, ^37^ Provided the central role of oxidative stress and inflammation in AD pathogenesis, we further evaluated the effects of exosomes on these key pathological processes. The Nrf2/HO‐1 signaling axis is a master regulator of cellular antioxidant defense, mitochondrial function, and inflammatory suppression.^5^, ^38^, ^39^ Although acute oxidative stress can activate this pathway, sustained pathological stress, as observed in AD, often results in impaired Nrf2 signaling, thereby exacerbating neuronal vulnerability. Exosomes treatment significantly enhanced nuclear translocation of Nrf2 and upregulated HO‐1 expression, as confirmed by immunocytochemical analysis. These findings suggest that exosomes restore redox homeostasis by reactivating endogenous antioxidant defense mechanisms while potentially suppressing pro‐inflammatory signaling pathways as further evidenced by evaluation of anti‐inflammatory activity of exosomes.

Neuroinflammation is another hallmark of AD and is marked by elevated levels of pro‐inflammatory cytokines, comprising TNF‐α and IL‐6, which drive neuronal dysfunction and disease progression. These cytokines are known to enhance BACE1 activity and NF‐κB signaling, thereby promoting amyloidogenic processing and exacerbating neurodegeneration.^3^, ^40^, ^41^ Similar to earlier studies,^42^, ^43^, ^44^ our results demonstrated significantly elevated TNF‐α and IL‐6 levels in STZ‐treated cells, confirming the presence of a pronounced inflammatory response. However, treatment with exosomes significantly reduced the levels of these cytokines, indicating a potent anti‐inflammatory effect. This reduction suggests that exosomes modulate cytokine production and restore immunological balance, thereby contributing to neuronal protection. Additionally, it was observed that ME was significantly more potent in alleviating oxidative stress and inflammation as compared to NE.

By describing their bioactive cargo and related signaling pathways, future research should clarify the molecular mechanisms behind the neuroprotective, anti‐oxidative, and anti‐inflammatory properties of exosomes. To enhance their translational and therapeutic potential, exosome modification techniques must be optimized, brain targeting must be enhanced, and thorough safety and dose‐response studies must be conducted in pertinent neurodegenerative models.

Collectively, the present findings demonstrate that BMSC‐derived exosomes exert multifaceted neuroprotective effects by reducing oxidative stress, and attenuating neuroinflammation. The stimulation of the Nrf2/HO‐1 pathway and repression of pro‐inflammatory cytokines provide a plausible mechanistic basis for the observed neuroprotection. These results highlight the therapeutic potential of exosome‐based interventions as a promising, cell‐free strategy for targeting key pathogenic mechanisms underlying AD (Figure 8).

**Figure 8:**
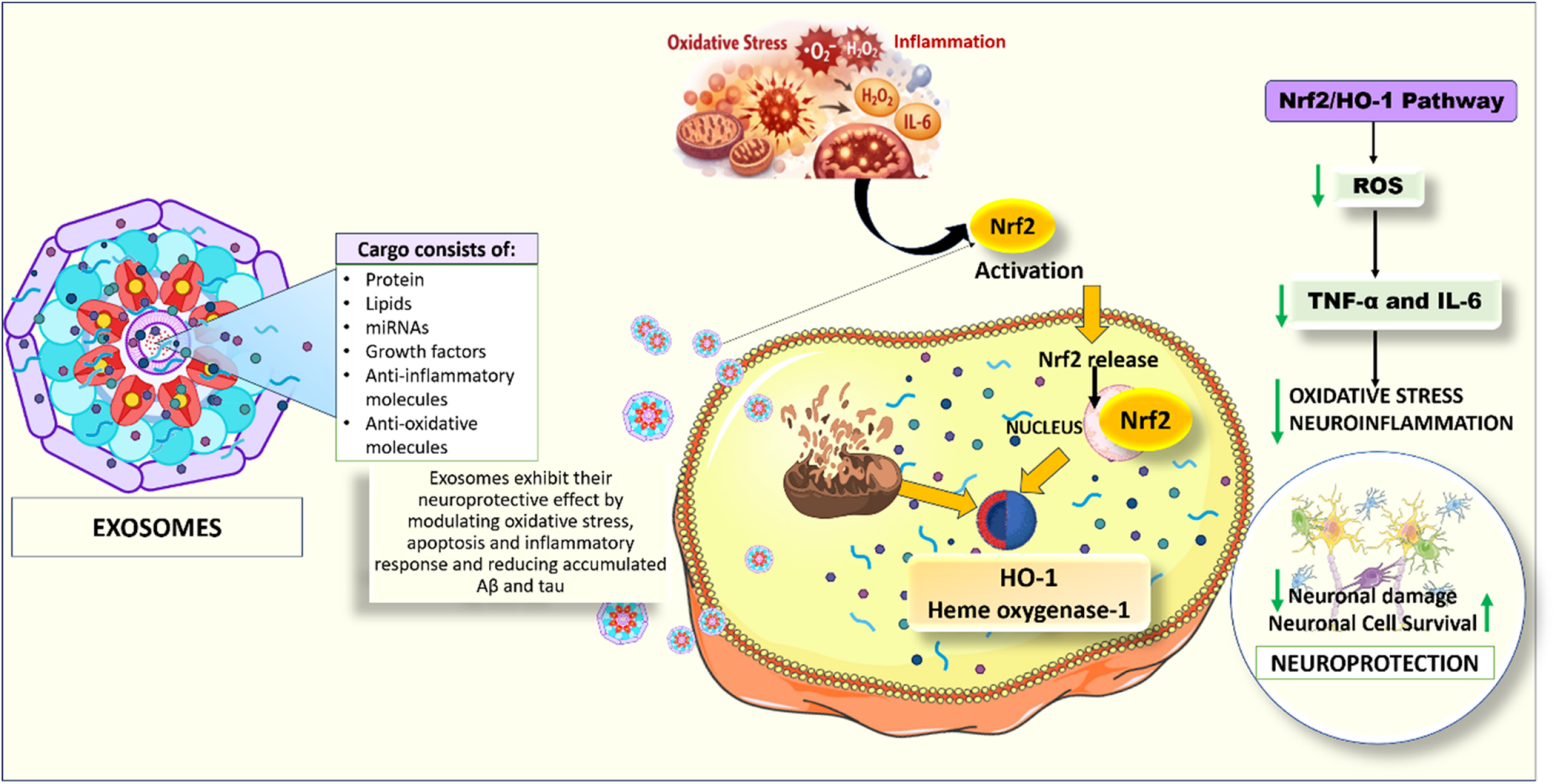
Neuroprotective mechanism of exosomes. Exosomes promote nuclear translocation of Nrf2, leading to upregulation of HO‐1 and antioxidant defenses. This results in attenuation of intracellular ROS generation and suppression of pro‐inflammatory cytokines, TNF‐α and IL‐6, thereby attenuating oxidative stress and neuroinflammation. Collectively, these effects contribute to enhanced neuronal survival and overall neuroprotection associated with exosomes.

## 5 CONCLUSION

In conclusion, the present study demonstrates that BMSCs‐derived exosomes exert pronounced antioxidative and anti‐inflammatory effects in an STZ‐induced SH‐SY5Y cellular model of AD, accompanied by enhanced neuronal survival and integrity. Exosome treatment effectively modulated the Nrf2/HO‐1 signaling axis, thereby restoring endogenous antioxidant defense mechanisms compromised under sustained oxidative stress conditions. The observed activation of this pathway underscores a key molecular mechanism through which ME confer neuroprotection. Collectively, our findings indicate that exosomes possess significant therapeutic potential by targeting multiple pathological hallmarks of neurodegeneration. At a concentration of 50 µg/mL, NE and ME robustly improved neuronal viability, restored neurotrophic support, attenuated oxidative damage, and suppressed pro‐inflammatory responses. The multimodal protective effects observed in this study highlight the capacity of exosomes to modulate interconnected pathogenic pathways underlying AD. Moreover, our results demonstrated that increased cell survival, as well as reduced oxidative stress and inflammation, indicated that ME was more therapeutically effective than NE in attenuating AD pathologies. These findings suggest that modification of exosomes increases their neuroprotective potential. Taken together, these results support the translational promise of exosomes as a novel, cell‐free therapeutic strategy for mitigating neurodegenerative processes associated with AD.

## AUTHOR CONTRIBUTIONS

Dibbanti HariKrishnaReddy and Balachandar Vellingiri designed the study. Rishika Dhapola, Prajjwal Sharma, and Sneha Kumari performed experimental work, data curation, and preparation of the manuscript. Rishika Dhapola, Prajjwal Sharma, and Sneha Kumari performed data analysis. Dibbanti HariKrishnaReddy and Balachandar Vellingiri revised the manuscript for important intellectual content. All authors read and approved the final manuscript. All persons designated as authors qualify for authorship, and all those who qualify for authorship are listed.

## CONFLICT OF INTEREST STATEMENT

The authors declare no conflicts of interest.

## ETHICS STATEMENT

All animal experiments were carried out in compliance with institutional policies for the care and management of animals, and the institutional animal ethics committee (IAEC) of Central University of Punjab authorized the study protocol (IAEC approval no. CUPB/IAEC/2022/53).

## Data Availability

The data that support the findings of this study are available on request from the corresponding author. The data are not publicly available due to privacy or ethical restrictions.
